# Effect of Cooking Methods on the Antioxidant Capacity of Plant Foods Submitted to In Vitro Digestion–Fermentation

**DOI:** 10.3390/antiox9121312

**Published:** 2020-12-21

**Authors:** Beatriz Navajas-Porras, Sergio Pérez-Burillo, Álvaro Jesús Valverde-Moya, Daniel Hinojosa-Nogueira, Silvia Pastoriza, José Ángel Rufián-Henares

**Affiliations:** 1 Centro de Investigación Biomédica, Departamento de Nutrición y Bromatología, Instituto de Nutrición y Tecnología de Alimentos, Universidad de Granada, 52005 Granada, Spain; beatriznavajas@ugr.es (B.N.-P.); spburillo@ugr.es (S.P.-B.); alvjvm@correo.ugr.es (Á.J.V.-M.); dhinojosa@ugr.es (D.H.-N.); spdelacueva@ugr.es (S.P.); 2 Instituto de Investigación Biosanitaria (ibs.GRANADA), Universidad de Granada, 52005 Granada, Spain

**Keywords:** antioxidant capacity, in vitro digestion–fermentation, thermal processing, cooking methods, plant foods

## Abstract

The antioxidant capacity of foods is essential to complement the body’s own endogenous antioxidant systems. The main antioxidant foods in the regular diet are those of plant origin. Although every kind of food has a different antioxidant capacity, thermal processing or cooking methods also play a role. In this work, the antioxidant capacity of 42 foods of vegetable origin was evaluated after in vitro digestion and fermentation. All foods were studied both raw and after different thermal processing methods, such as boiling, grilling roasting, frying, toasting and brewing. The cooking methods had an impact on the antioxidant capacity of the digested and fermented fractions, allowing the release and transformation of antioxidant compounds. In general, the fermented fraction accounted for up to 80–98% of the total antioxidant capacity. The most antioxidant foods were cocoa and legumes, which contributed to 20% of the daily antioxidant capacity intake. Finally, it was found that the antioxidant capacity of the studied foods was much higher than those reported by other authors since digestion–fermentation pretreatment allows for a higher extraction of antioxidant compounds and their transformation by the gut microbiota.

## 1. Introduction

According to nutritional epidemiological evidence, the consumption of fruit and vegetables, as well as other plant-derived foods, is associated with a protective effect against several noncommunicable diseases such as cardiovascular disease, diabetes 2, metabolic syndrome, cancer or inflammation, where oxidative stress plays an important role [1]. Phytochemicals, such as phenolic compounds, have been noted as partially responsible for such protective effects against chronic diseases [2]. These compounds are mainly responsible for the antioxidant capacity of plant-derived foods [3], which is related to their culinary treatment. Some vegetables, mostly fruits, are consumed in their raw form, whereas others are cooked before being eaten. There are many different culinary treatments, ranging from those that use water as a cooking medium to those that use oil [4]. Whereas cooking in water may result in the loss of hydrosoluble compounds (mainly vitamins and minerals), cooking in oil could result in an enrichment of phytochemicals, though this will depend on the oil used [5]. Additionally, different cooking methods use different temperatures, ranging from 60 to 70 °C in steaming to 200 to 220 °C in grilling. Higher temperatures will have a deeper impact on thermolabile compounds and therefore reduce their availability. However, it has been seen that higher temperatures or intense cooking achieve a deeper breakdown of the plant cell wall, increasing the availability of some compounds, and therefore making them easier to absorb [6]. On the other hand, thermal processing can cause chemical changes in plant foods’ composition due to the development of the Maillard reaction, triggered by the interaction of carbonyl compounds with amino groups [7]. Therefore, a long thermal processing time in cooking could result in the generation of potentially toxic chemical species [8] and loss of bioactive compounds [9].

On the other hand, once ingested, foods undergo gastrointestinal digestion. However, due to the lack of enzymes to digest plant cell wall polysaccharides, some structures remain relatively intact upon reaching the large intestine. It is well known that fiber is the main energy source for gut microbes [10] and phenolic compounds are mainly absorbed in the large intestine after extensive metabolization by gut bacteria [11]. Therefore, most antioxidants from vegetables are absorbed in the large intestine. 

In order to shed some light on this matter, this paper evaluates the antioxidant capacity of the most representative food items from the vegetable kingdom submitted to different cooking methods (boiling, grilling, roasting, frying and toasting). In addition, these foods were submitted to in vitro digestion–fermentation to simulate the activity of the human gut microbiota for a more realistic estimation of the antioxidant capacity of foods. Finally, the contribution of the intake of plant foods to the daily antioxidant capacity of the Spanish diet was determined.

## 2. Materials and Methods 

### 2.1. Chemicals

#### 2.1.1. *In Vitro* Digestion and Fermentation

Salivary alpha-amylase, pepsin, porcine bile acids (porcine bile extract), sodium dihydrogen phosphate, sodium sulfide, resazurin, tryptone and cysteine were obtained from Sigma-Aldrich (Darmstadt, Germany). Pancreatin (from porcine pancreas) was purchased from Alpha Aesar (Kandel, Germany). 

#### 2.1.2. Antioxidant Capacity

2,2 Diphenyl-1-1-picrythydrazul hydrate 95% (DPPH), Trolox ((±)-6-hydroxy-2,5,7,8-tetramethylchromane-2-carboxylic acid), 2,4,6-tri(2-pyridyl)-s-triazine (TPTZ), sodium acetate, iron (III) chloride hexahydrate, methanol, hydrochloric acid, sodium carbonate, gallic acid and the Folin–Ciocalteu® reagent were purchased from Sigma-Aldrich (Darmstadt, Germany). 

### 2.2. Plant Foods and Cooking Conditions

A total of 42 plant foods (Table S1) were studied, belonging to the following groups: alcoholic drinks (beer, red wine), cereals (regular biscuits, whole-grain biscuits, bread, whole-grain bread, breakfast cereals, whole-grain breakfast cereals, penne, whole-grain penne, rice, whole-grain rice), cocoa (dark chocolate, Nutella), coffee (regular coffee, instant coffee), fruits (apple, banana, grape, olive, orange, peach, plum), legumes (kidney beans, lentils), nuts (nuts mixture, peanuts), oils (olive oil, sunflower oil), tubers (potato, sweet potato) and vegetables (cabbage, carrot, cauliflower, eggplant, lettuce, onion, pepper, spinach, tomato, zucchini). Samples were obtained from three different retail stores and stored under refrigeration (fresh vegetables) or at room temperature (according to the manufacturer’s instructions) for a maximum of 3 days before cooking. The regular coffee brew was prepared from commercially roasted coffee (100% Arabica) supplied by a national producer. The coffee brew was prepared with a mocha-type domestic coffee pot with 62.5 g of coffee per 1000 mL of water. The soluble coffee brew (100% Arabica from a national producer) was prepared following the manufacturer’s instructions (2.0 g of soluble coffee per 100 mL of boiling water).

The samples were submitted to different culinary treatments: boiling, grilling, roasting, frying, and toasting. Brewing was also included as a culinary treatment (as stated above for coffees), although it involves a technological process to obtain some of the liquid foods studied (beer and red wine). In addition, for some samples, the raw food was also investigated as this is the common way to eat those foods. A total of 107 samples were obtained. Fruits, vegetables and tubers were cut in different sizes to achieve the same final texture after the different thermal treatments were applied.

Extra virgin olive oil (EVOO) was used as a cooking medium for grilling and frying. Boiling was performed at 100 °C for 20 min at a water/food rate of 5:1. Grilling was performed at 220–250 °C for 3 min on each side at an oil/food rate of 0.5:1. Roasting was performed at 180 °C for 10 min. Fried foods were obtained at 180 °C for 8 min at an oil/food rate of 5:1. Toasting was performed in a Grunkel TS140H toaster at the fourth level for 3 min at 900 W following the manufacturer’s instructions. Cooking times and food/medium rates were acquired from Ramírez-Anaya et al. [5] and adapted to our equipment and laboratory conditions. Samples and treatments are listed in Table S1. 

The utensils used for sample preparation were the following: stainless steel spoons, forks, and knives; saucepan, frying pan, a portable oven (1500 W), fryer, and toaster. All these utensils were purchased from Centro Hogar Sánchez (Granada, Spain). Samples were homogenized and stored under a nitrogen atmosphere at −80 °C in order to avoid oxidation. All analyses were performed in duplicate.

### 2.3. In Vitro Gastrointestinal Digestion and Fermentation

Plant foods, after proper cooking, were submitted to in vitro batch digestion–fermentation in order to mimic physiological processes in the human gut, according to a protocol previously described [12]. For each sample, 5 g of food was submitted (in triplicate) to in vitro gastrointestinal digestion followed by in vitro fermentation. The food was added to falcon tubes along with three phases: oral, gastric and intestinal. The oral digestion phase was performed with α-amylase for 2 min under agitation at 37 °C. The gastric phase was performed with pepsin for 2 h with agitation at pH 2–3 ay 37 °C. The gastric phase was performed with bile salts and pancreatin for 2 h under agitation at pH 7 at 37 °C. In vitro fermentation was performed at 37 °C for 24 h using fecal samples from five healthy donors (mean body mass index = 21.3; no antibiotics taken for three months prior to the assay). The fecal samples were pooled together to reduce interindividual variability. The samples were then centrifuged, and the supernatants were taken for analysis. A control fermentation was performed using only the fecal fermentation solution (inoculum composed of peptone, cysteine, and resazurin). 

After in vitro gastrointestinal digestion and fermentation, two fractions were obtained: a digested fraction (available for absorption at the small intestine) and a fermented fraction (available for absorption at the large intestine). 

### 2.4. Antioxidant Assays 

The antioxidant capacity was evaluated in the two fractions obtained after in vitro digestion and fermentation: the supernatant obtained after gastrointestinal digestion (potentially absorbable in the small intestine) and the supernatant obtained after fermentation (potentially absorbable in the large intestine). The sum of the two fractions accounts for the total antioxidant activity that each food could exert within the human body [13]. Three different methods were used to determine antioxidant capacity (DPPH, FRAP and Folin–Ciocalteu). All the antioxidant capacity values of the three methods were corrected for their respective blanks (enzymes, chemicals and inoculum).

Trolox equivalent antioxidant capacity against DPPH radicals (TEAC_DPPH_) assay. This method was conducted according to the procedure of Yen and Chen [14] on a microplate reader (FLUOStar Omega, BMG Labtech, Ortenberg, Germany). Briefly, 20 μL of either digestion or fermentation supernatants was added to a 96-well plate in duplicate and mixed with 280 μL of DPPH reagent (74 mg DPPH/L methanol). The antioxidant reaction was monitored at 37 °C for 60 min. The calibration curve was prepared with Trolox in the range of 0.01–0.4 mg/mL. Results were expressed as mmol Trolox equivalent/kg of food. 

Folin–Ciocalteu assay. The method was conducted as described by Moreno-Montoro and colleagues [15] on a microplate reader (FLUOStar Omega, BMG Labtech, Ortenberg, Germany). Briefly, 30 μL of either digestion or fermentation supernatants was added in duplicate to a 96-well plate and mixed with 15 μL of Folin–Ciocalteu reactive, 190 μL of distilled water and 60 μL of 10% sodium carbonate solution. The antioxidant reaction was monitored at 37 °C for 30 min. The calibration curve was prepared with gallic acid in the range of 0.1–2.5 mg/mL. Results were expressed as mg gallic acid equivalent/kg of food.

Trolox equivalent antioxidant capacity referred to reducing capacity (TEAC_FRAP_) assay. The ferric reduction capacity of samples was assessed through the procedure described by Benzie and Strain [16] on a microplate reader (FLUOStar Omega, BMG Labtech, Ortenberg, Germany). Briefly, 20 μL of either digestion or fermentation supernatants was added to a 96-well plate, in duplicate, and mixed with 280 μL of FRAP reagent (freshly prepared each day). The antioxidant reaction was monitored at 37 °C for 30 min. The calibration curve was prepared with Trolox in the range of 0.01–0.4 mg/mL. Results were expressed as mmol Trolox equivalent/kg of food. 

### 2.5. Calculations of Daily Antioxidant Intake

The individual contribution of each food group to the dietary antioxidant capacity intake was calculated, taking into account their daily consumption and the amount of food per serving [17] as well as the antioxidant capacity previously measured for the samples. The antioxidant capacity of each food referred to the usual serving size in Spain [18] and was compared with the results previously published by Saura-Calixto and Goñi [19].

### 2.6. Statistical Analysis

The statistical significance of the data was tested by one-way analysis of variance (ANOVA), followed by the Duncan test to compare the means that showed a significant variation (*p <* 0.05). As a factor for ANOVA, we used the type of cooking (boiled, brewed, fried, grilled, raw, roasted and toasted), type of food (alcoholic drinks, cereals, cocoa, coffee, fruits, legumes, nuts, oils, tubers and vegetables) and type of sample (cereals: biscuits, bread, breakfast cereals, penne and rice; fruits: apple, banana, grapes, olives, orange, peach and plum; vegetables: cabbage, carrot, cauliflower, eggplant, lettuce, onion, pepper, spinach, tomato and zucchini). Statistical analysis was performed using raw vegetables and the mean of all food groups as the reference groups. Pearson’s correlation coefficient was calculated to show the linear relation between antioxidant capacity at *p <* 0.05. To obtain the significance between the different levels within the same group, Tukey’s test was performed. All statistical analyses were performed using Statgraphics Plus software, version 5.1. 

## 3. Results 

We tested the potential physiological antioxidant capacity of plant foods after in vitro digestion–fermentation with three different methods (DPPH, FRAP and Folin–Ciocalteu). In general, a linear correlation was obtained by the Spearman method between the three methods (Figure S1). The significant correlations found (*p <* 0.05) were positive, with values around Rs = 0.80. 

In the following sections, a deeper description of the results obtained by type of cooking, type of food and each group of plant foods will be reported.

### 3.1. Samples by Type of Cooking

Cooking methods had an impact on the antioxidant capacity of the digested fraction, as will be explained in the following sections.

#### 3.1.1. Gastrointestinal Digested Fraction

**Figure 1:**
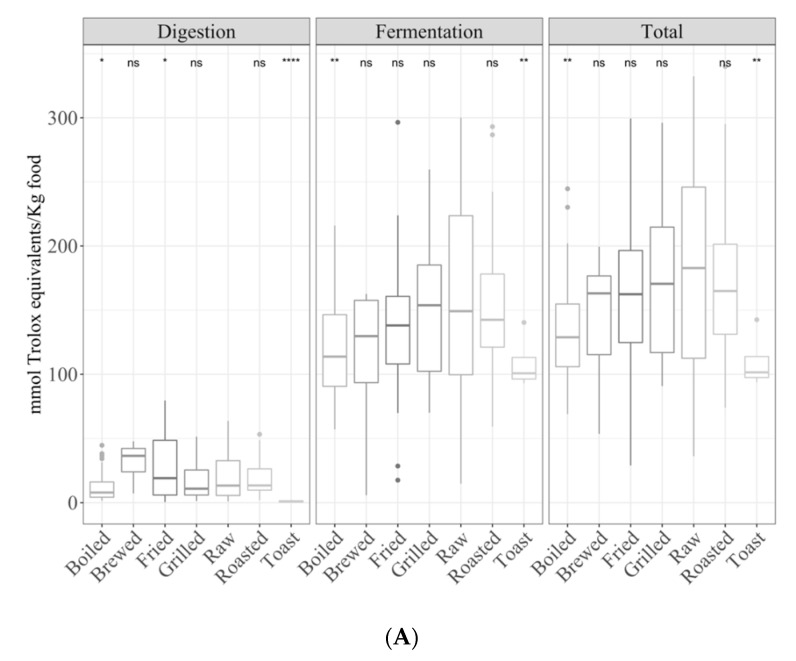

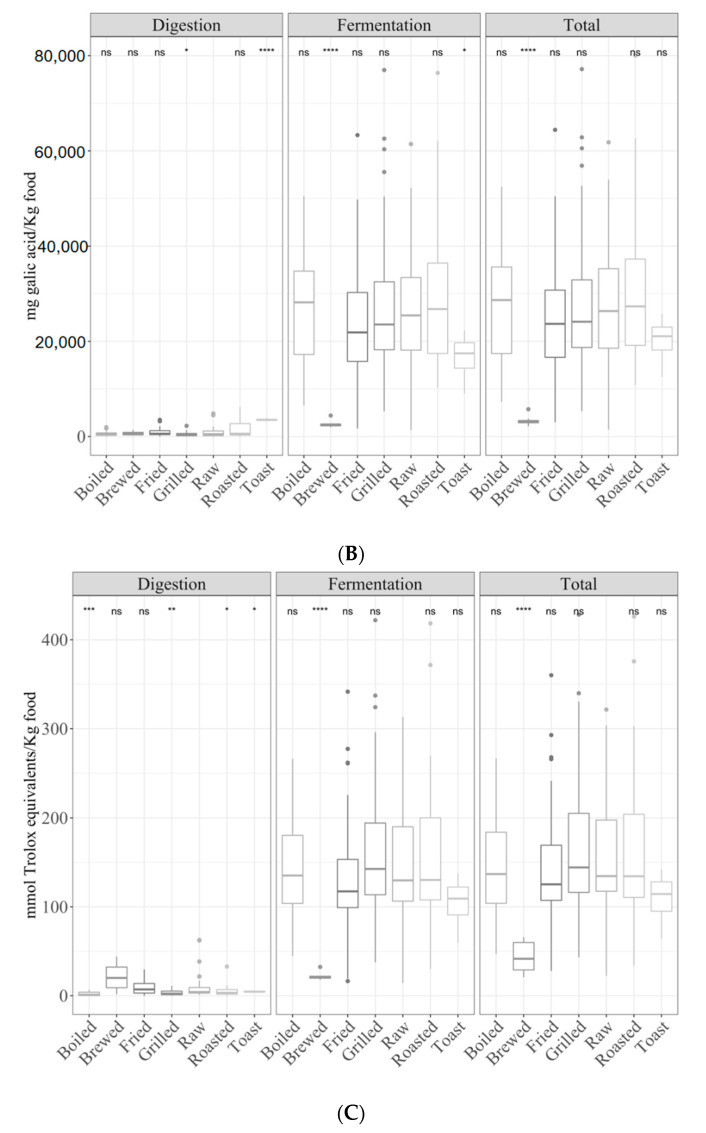
Antioxidant capacity of plant foods (obtained after *in vitro* digestion and fermentation) depending on the cooking technique (**A**) Trolox equivalent antioxidant capacity against DPPH radicals (TEAC_DPPH_), (**B**) Folin–Ciocalteu and (**C**) for Trolox equivalent antioxidant capacity referred to reducing capacity (TEAC_FRAP_)). Statistical analysis was performed via ANOVA using raw vegetables as the reference group. Statistic labels: *: *p <* 0.05, **: *p <* 0.01, ***: *p <* 0.001, ****: *p <* 0.0001, ns: not significant.

Raw foods showed a significantly (*p <* 0.05) higher antioxidant capacity than boiled and toasted foods for the TEAC_DPPH_ assay (Table S2) but lower than fried foods (Figure 1A). In the case of the Folin–Ciocalteu method, we observed a significantly higher antioxidant capacity in toasted foods vs. raw foods (*p <* 0.05) as opposed to the previous method; however, raw foods were still more antioxidant than those cooked with the other methods (Figure 1B). For the TEAC_FRAP_ method, raw foods were more (*p <* 0.05) antioxidant than boiled, grilled and toasted foodstuffs, as in the other methods (Figure 1C).

Comparing the mean antioxidant capacity of digested samples after different cooking treatments, the following significant differences were found (ANOVA paired comparison; *p <* 0.05): for the TEAC_DPPH_ method, fried foods were more antioxidant than boiled; for the Folin–Ciocalteu method, toasting resulted in higher antioxidant values than those of the other cooking methods; for the TEAC_FRAP_ method, raw foods showed higher antioxidant capacity than grilled foods. 

#### 3.1.2. Gastrointestinal Fermented Fraction

Microbial fermentation has a deep impact on food antioxidant capacity [12] since the gut microbiota is able to metabolize those undigested nutrients reaching the large intestine and release many metabolites with a potent antioxidant capacity. With the TEAC_DPPH_ method, the antioxidant capacity of the fermented fraction was significantly (*p <* 0.05) lower in boiled and toasted vegetables compared to raw foods (Figure 1A). On the other hand, the Folin–Ciocalteu method showed a significantly (*p <* 0.05) lower antioxidant capacity in brews and toasted foods compared to raw foods (Figure 1B). The TEAC_FRAP_ results were on the same line (Figure 1C). 

#### 3.1.3. Total Antioxidant Capacity

In general, the contribution of the digested fraction to the total antioxidant capacity was much lower than that of the fermented fraction since many different bioactive compounds could be released from the food matrix by the microbial activity in addition to a potential generation of new antioxidant metabolites. 

**Figure 2:**
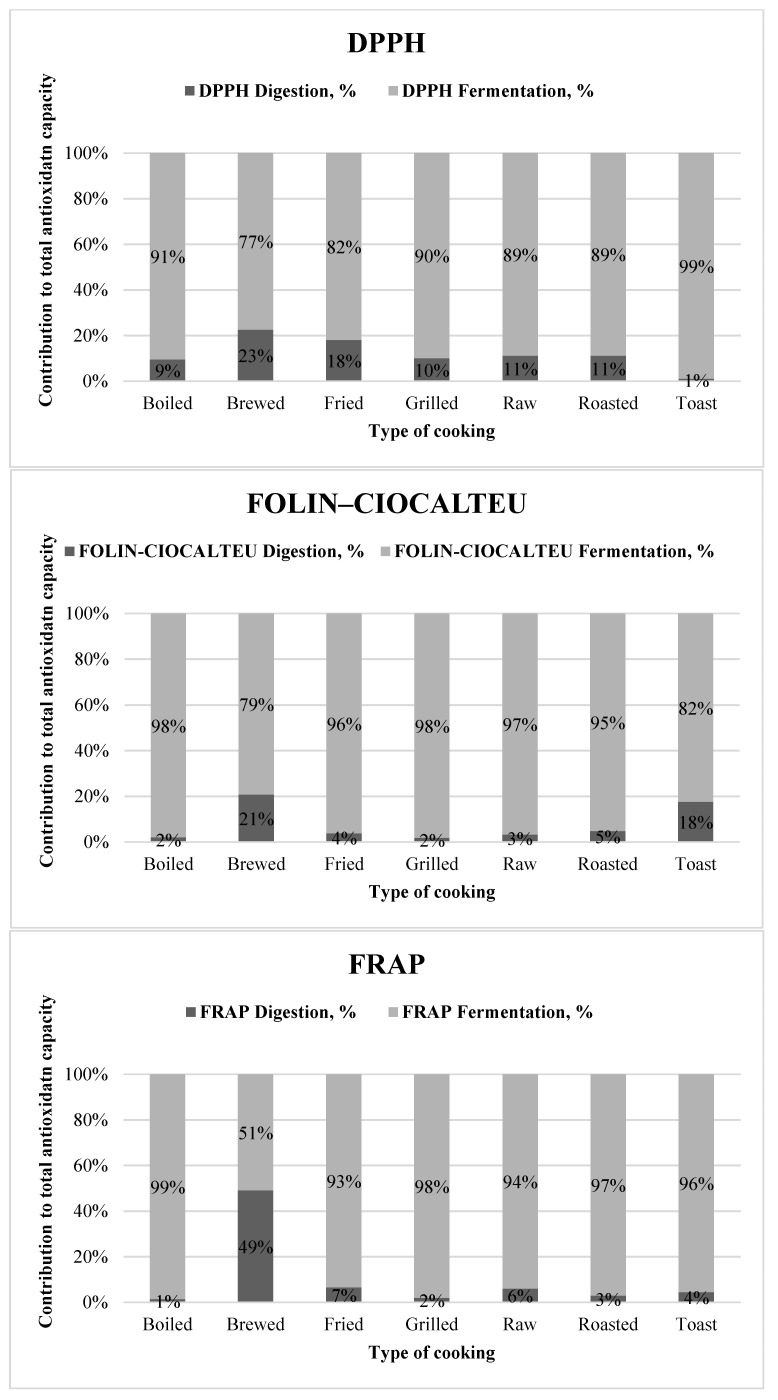
Contribution to the total antioxidant capacity of each fraction depending on the cooking technique.

Similarly to the fermented fraction, the total antioxidant capacity of boiled and roasted foods was lower (*p <* 0.05) than that of raw foods (Figure 1A). Overall, for most of the cooking techniques, the contribution of the digested fraction to the total antioxidant capacity was around 10% (Figure 2) but for brewed foods (accounted for 23%) and toasted plant foods (just 1%). 

The Folin–Ciocalteu method showed also a significantly (*p <* 0.05) lower total antioxidant capacity for brewed and toasted foods (Figure 1B). In this case, however, the digestion fraction had a lower contribution to the total antioxidant capacity for most of the cooking techniques (around 4%), compared to DPPH. The same results were obtained for the FRAP method (Figure 1C and Figure 2). For this method, it is noteworthy to mention that although the digested fraction contributed very little to the total reducing capacity (around 3%), in the case of brews, 50% of the antioxidant capacity was obtained for both fractions (Figure 2). 

### 3.2. Samples by Type of Food

The type of plant had an important effect on the antioxidant capacity since the food matrix and composition are different. 

#### 3.2.1. Gastrointestinal Digested Fraction

**Figure 3:**
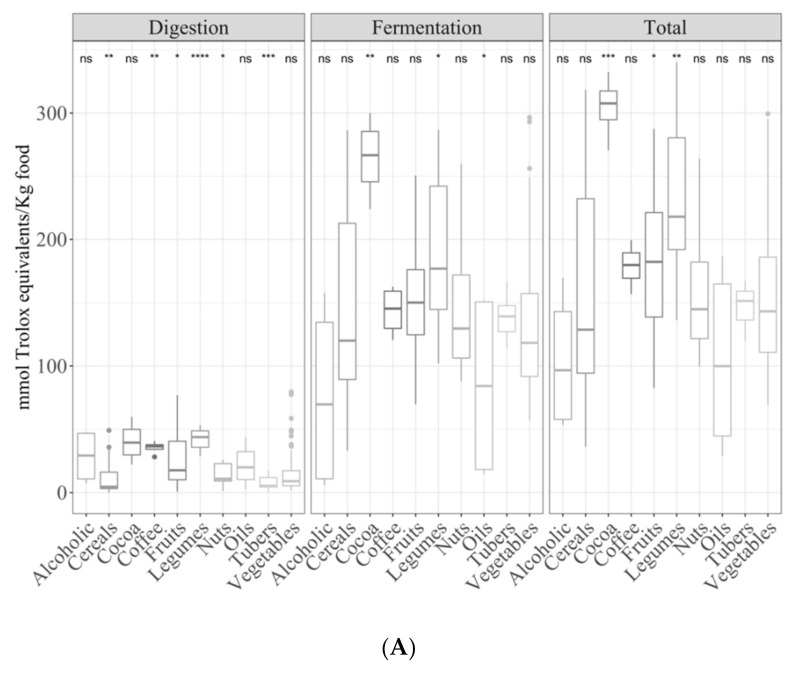

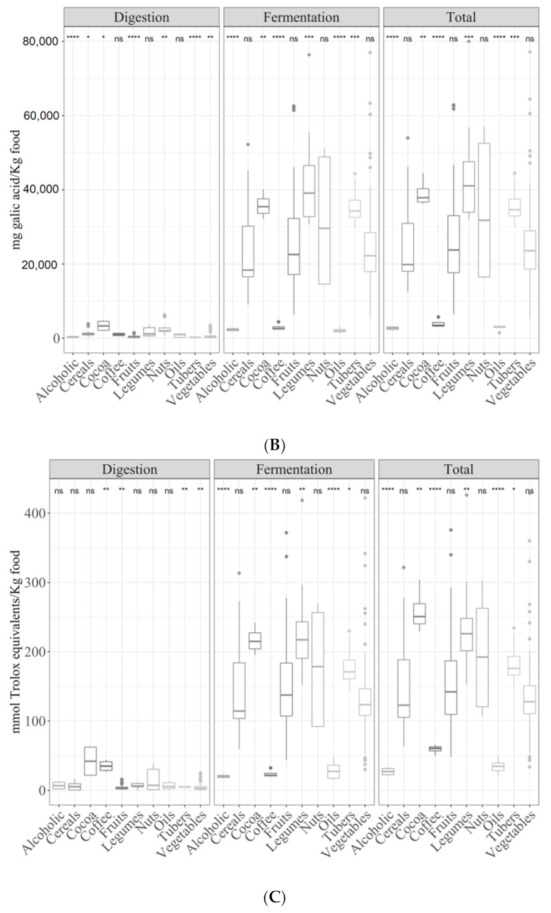
Antioxidant capacity of plant foods (obtained after *in vitro* digestion and fermentation) depending on the food group ((**A**) TEAC_DPPH_, (**B**) Folin–Ciocalteu and (**C**) TEAC_FRAP_). Statistical analysis was performed via ANOVA using the mean antioxidant capacity of all food groups as the reference group. Statistic labels: *: *p <* 0.05, **: *p <* 0.01, ***: *p <* 0.001, ****: *p <* 0.0001, ns: not significant.

Compared with the mean antioxidant capacity of all plant foods, coffee, fruits and legumes had a significantly higher antioxidant capacity (*p <* 0.05) for the TEAC_DPPH_ method (Table S3); however, lower values were obtained for cereals, nuts and tubers (Figure 3A). On the other hand, the Folin–Ciocalteu method showed a higher (*p <* 0.05) antioxidant capacity for nuts, tubers, alcoholic drinks, fruits and vegetables (Figure 3B). Regarding reducing capacity (TEAC_FRAP_), it was higher (*p <* 0.05) for coffee but lower for tubers, fruits and vegetables (Figure 3C).

When comparisons were made between different types of plant foods (ANOVA paired comparison; *p <* 0.05), the following significant differences were found: for the TEAC_DPPH_ method, fruits had a higher antioxidant capacity than cereals and vegetables, whereas legumes were more antioxidant than cereals, nuts, tubers and vegetables. In the case of the Folin–Ciocalteu method, cocoa and nuts had a higher mean than the rest of the foods (with no significant differences between them), and fruits were more antioxidant than cereals. Finally, for the TEAC_FRAP_ method, cocoa and coffee were more antioxidant than the other groups.

#### 3.2.2. Gastrointestinal Fermented Fraction

Cocoa and legumes had a stronger antioxidant capacity against DPPH radicals (*p <* 0.05) than the other food groups (Figure 3A). On the other hand, Folin–Ciocalteu showed a significantly (*p <* 0.05) higher antioxidant capacity for cocoa, legumes and tubers (Figure 3B). The TEAC_FRAP_ results followed the same tendency as the Folin–Ciocalteu method (Figure 3C).

#### 3.2.3. Total Antioxidant Capacity

When the total antioxidant capacity was studied grouped by type of food, cocoa and legumes were the most antioxidant foods (*p <* 0.05; Figure 3A), reaching up to 305 and 229 mmol Trolox/kg, respectively. As for the fermented fraction, in the case of the Folin–Ciocalteu method, cocoa, legumes and tubers were the most antioxidant foods (Figure 3B). The same results were observed for the FRAP method (Figure 3C). 

### 3.3. Detailed Analysis of Large Food Groups

Data on the digested and fermented fractions, as well as the total antioxidant capacity of those samples from larger groups (cereals, fruits and vegetables), were also analyzed separately. 

#### 3.3.1. Cereals

**Figure 4:**
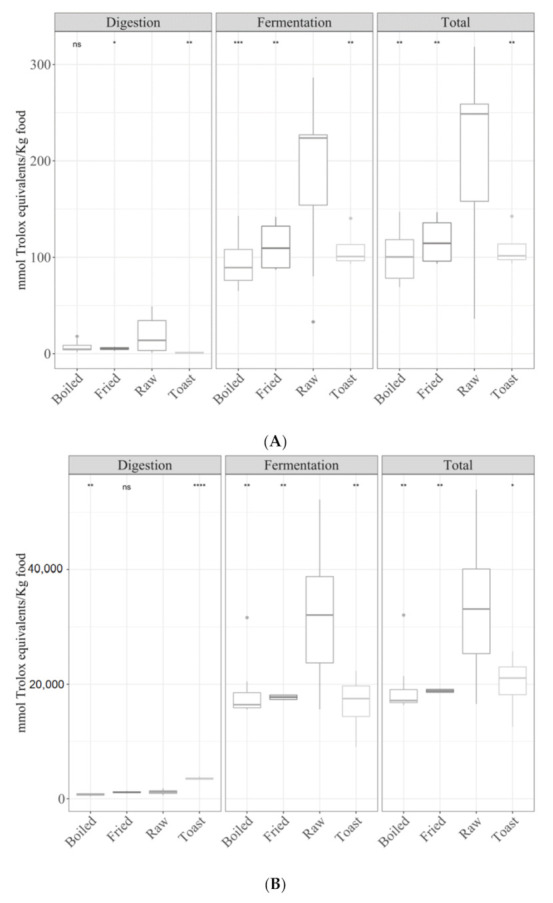

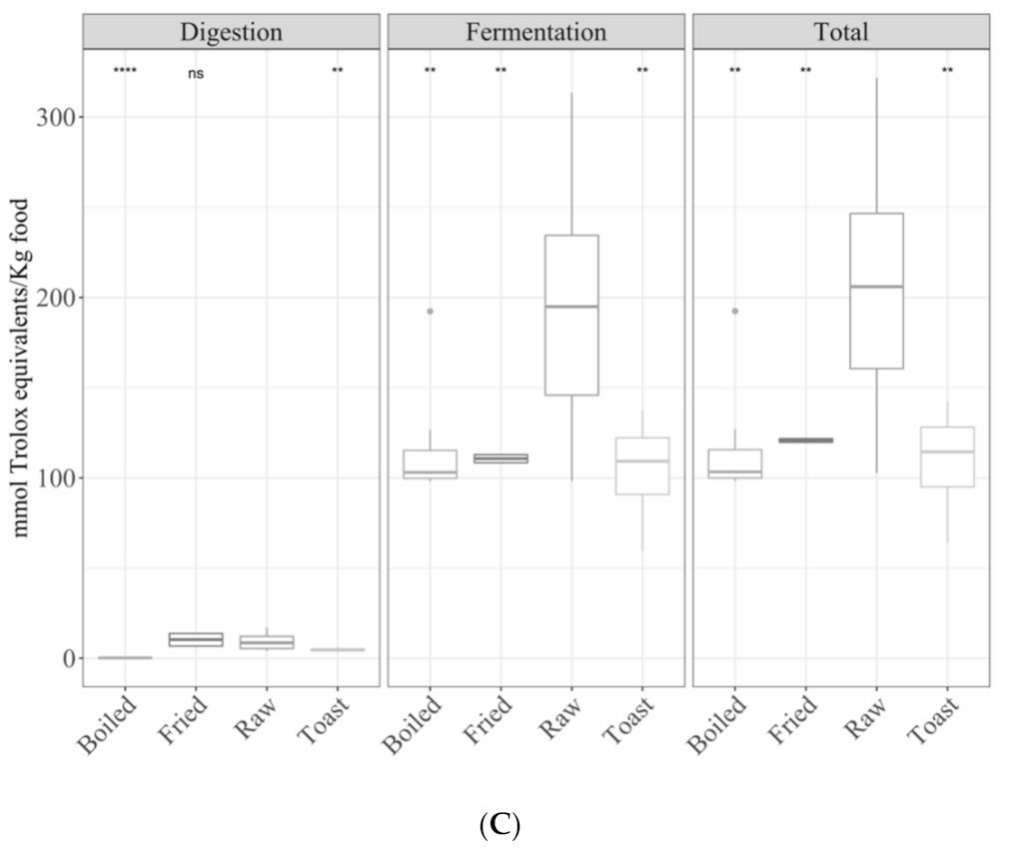
Antioxidant capacity of digested-fermented cereals depending on the type of cooking technique ((**A**) TEAC_DPPH_, (**B**) Folin–Ciocalteu and (**C**) TEAC_FRAP_). Statistical analysis was performed via ANOVA using raw cereals as the reference group. Statistic labels: *: *p <* 0.05, **: *p <* 0.01, ***: *p <* 0.001, ****: *p <* 0.0001, ns: not significant.

Regarding the effect of cooking methods on the antioxidant capacity of cereals, the mean antioxidant capacity of raw cereals (ANOVA paired comparisons, *p <* 0.05) measured with TEAC_DPPH_ was higher than that of the other cooking methods for both the digested and fermented fractions, resulting in a higher total antioxidant capacity (Figure 4A). In the case of the Folin–Ciocalteu method (Figure 4B), the antioxidant capacity of the digested fraction decreased as follows: toasted > raw > boiled. However, for the fermented fraction and total antioxidant capacity, raw cereals were the most antioxidant foodstuffs (*p <* 0.05). Finally, the reducing capacity of cooked cereals (TEAC_FRAP_) showed the same behavior as the DPPH method (Figure 4C). 

**Figure 5:**
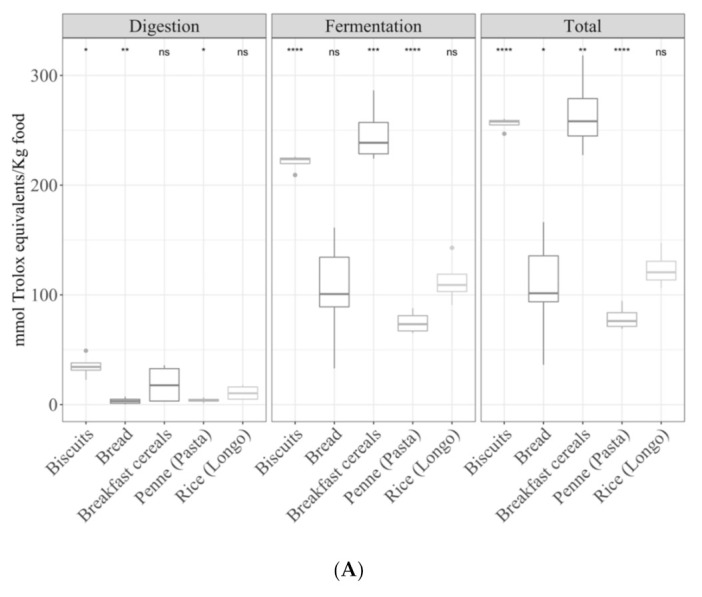

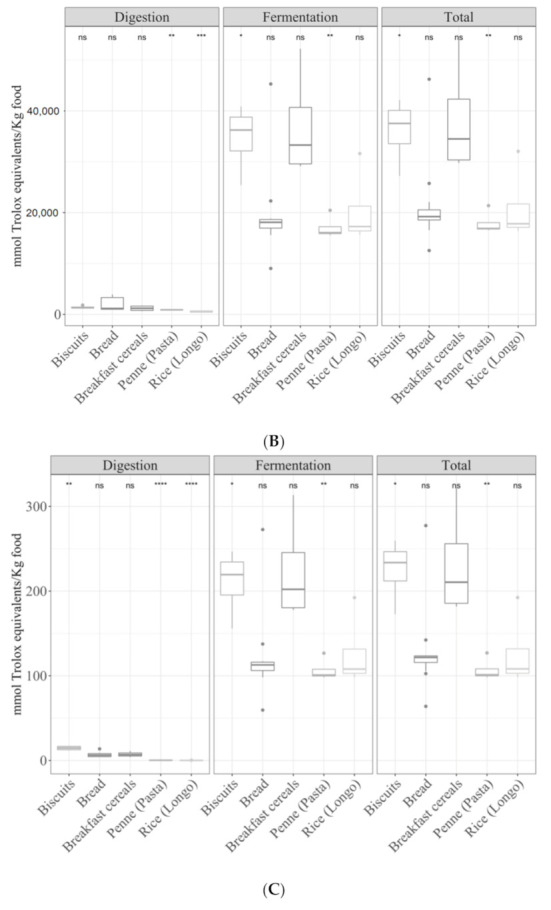
Antioxidant capacity of digested-fermented cereals depending on the sample type ((**A**) TEAC_DPPH_, (**B**) for Folin–Ciocalteu and (**C**) TEAC_FRAP_). Statistical analysis was performed through ANOVA using the mean of all food groups as the reference value. Statistic labels: *: *p <* 0.05, **: *p <* 0.01, ***: *p <* 0.001, ****: *p <* 0.0001, ns: not significant.

On the other hand, when samples were compared, depending on the type of cereal-based food, they behaved similarly irrespective of the antioxidant assay (Figure 5A–C). For the digested fraction, biscuits were more antioxidant (*p <* 0.05) than the other foods. However, for the fermented fraction and total antioxidant capacity, the following order was obtained: biscuits = breakfast cereals > bread = rice = pasta. Comparisons were also made between refined and whole-grain cereal products, but no significant differences were observed. 

#### 3.3.2. Fruits

**Figure 6:**
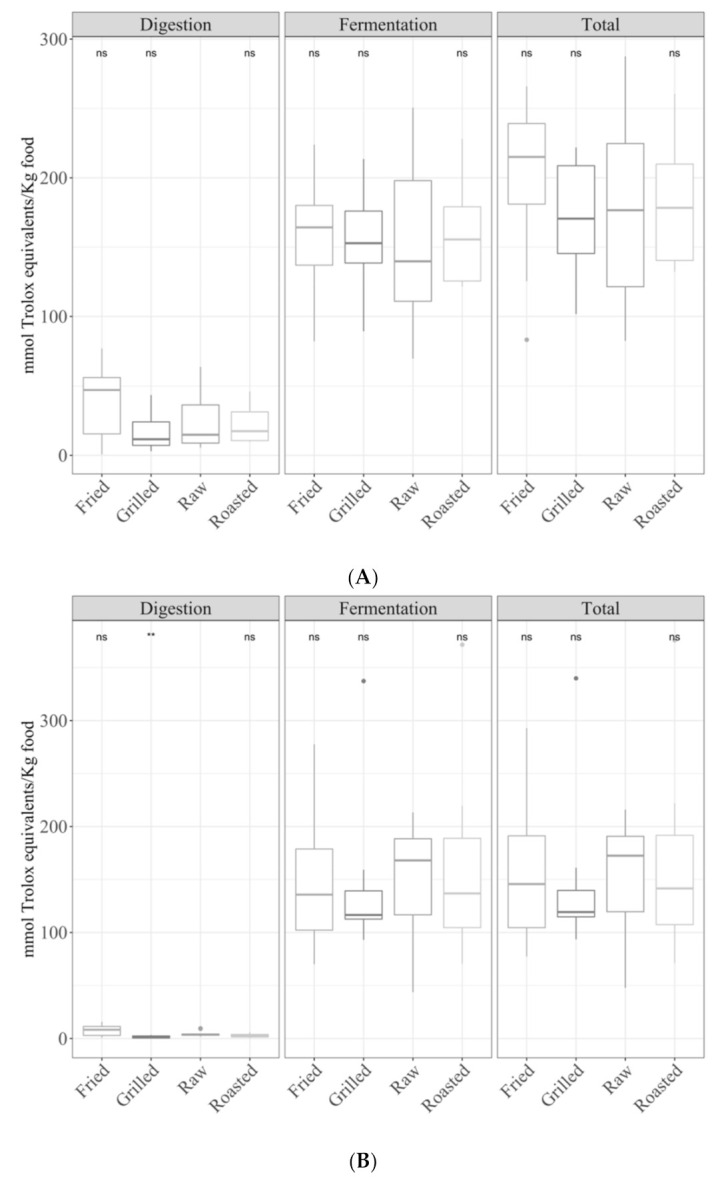

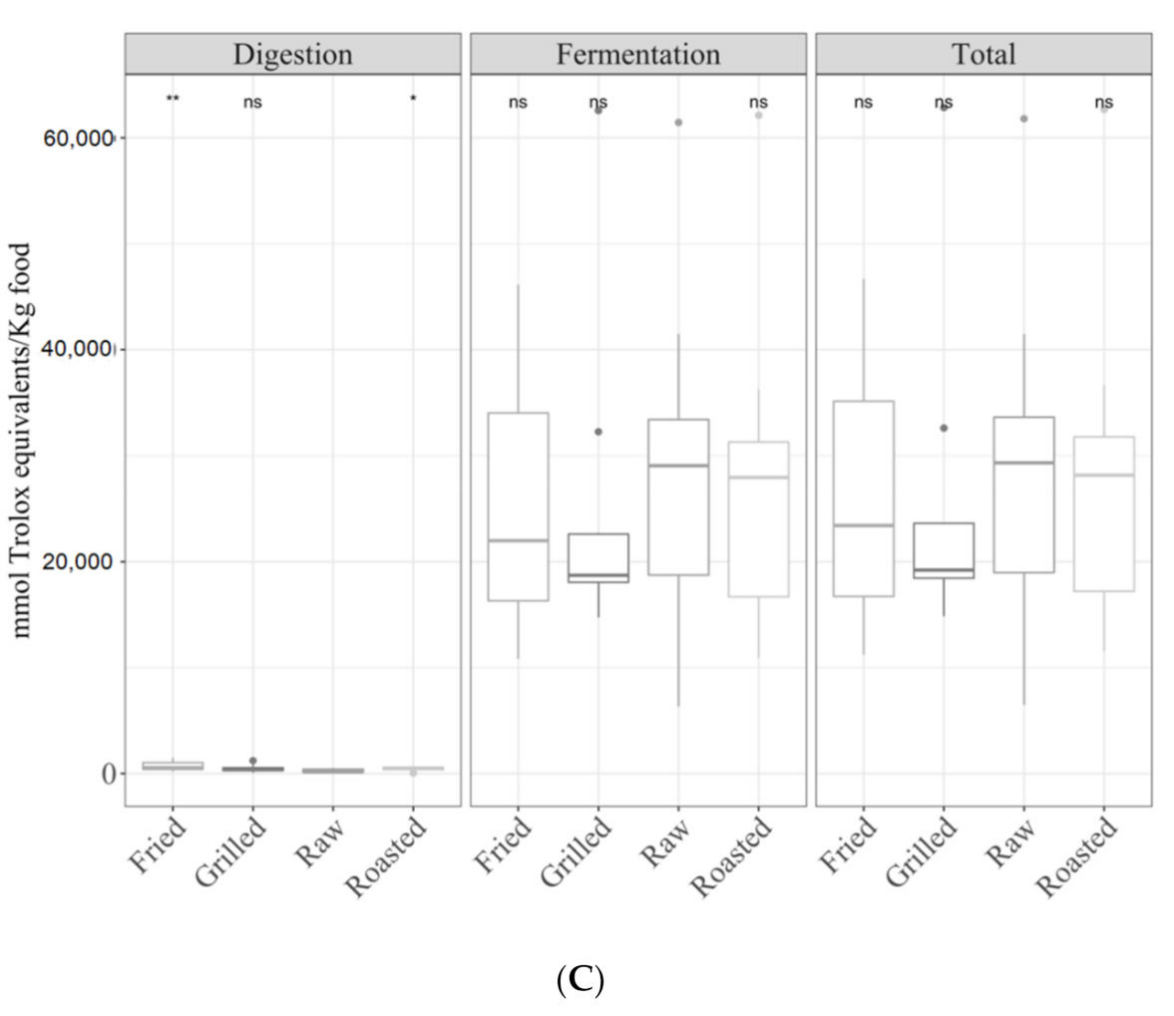
Antioxidant capacity of digested-fermented fruits depending on the type of cooking technique ((**A**) TEAC_DPPH_, (**B**) Folin–Ciocalteu and (**C**) TEAC_FRAP_). Statistical analysis was performed via ANOVA using raw fruits as the reference group. Statistic labels: *: *p <* 0.05, **: *p <* 0.01, ns: not significant.

Although fruits are usually consumed in raw form, they were submitted to different cooking techniques since some are heat-treated, especially for some desserts. In the digested fractions, fried and roasted fruits were more antioxidant (*p <* 0.05) than raw and grilled foods but only for the Folin–Ciocalteu method (Figure 6B). No statistically significant differences were observed for the TEAC_DPPH_ (Figure 6A) and TEAC_FRAP_ assays (Figure 6C). No significant differences among the four cooking techniques were observed for the fermented fraction or total antioxidant capacity.

**Figure 7:**
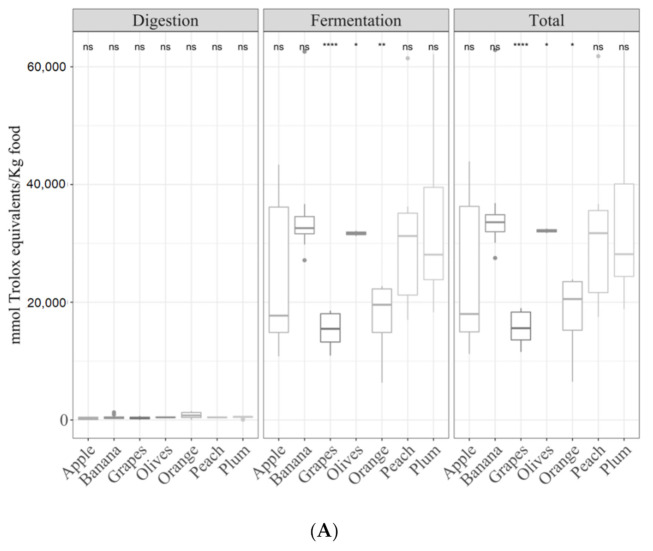

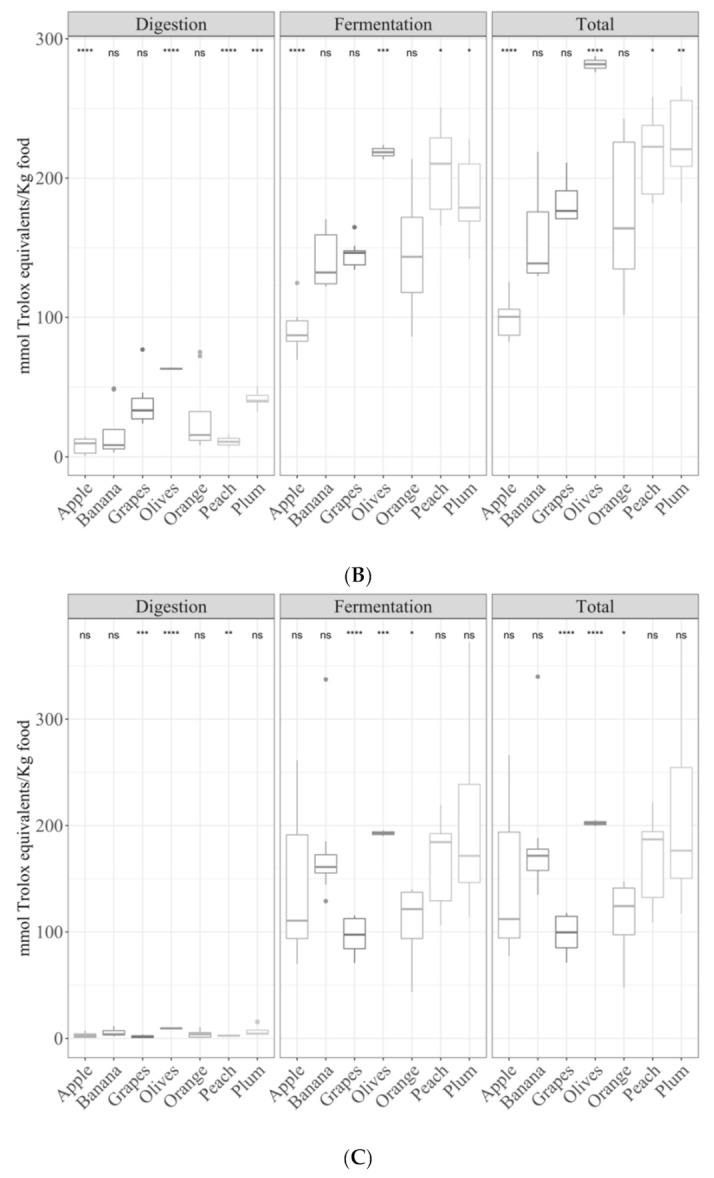
Antioxidant capacity of digested-fermented fruits depending on sample type ((**A**) TEAC_DPPH_, (**B**) Folin–Ciocalteu and (**C**) for TEAC_FRAP_). Statistical analysis was performed via ANOVA using the mean of all food groups as the reference group. Statistic labels: *: *p <* 0.05, **: *p <* 0.01, ***: *p <* 0.001, ****: *p <* 0.0001, ns: not significant.

Seven different fruits (apple, banana, grape, olive, orange, peach and plum) were analyzed. The digested fraction of olives and plum were more antioxidant (*p <* 0.05) than the other fruits but just for the TEAC_DPPH_ method (Figure 7A). For the fermented fraction and total antioxidant capacity, the following order was obtained: olives > peach = plum > grape = orange = banana = apple for the TEAC_DPPH_ (Figure 7A), Folin–Ciocalteu (Figure 7B) and TEAC_FRAP_ (Figure 7C) methods. 

#### 3.3.3. Vegetables 

**Figure 8:**
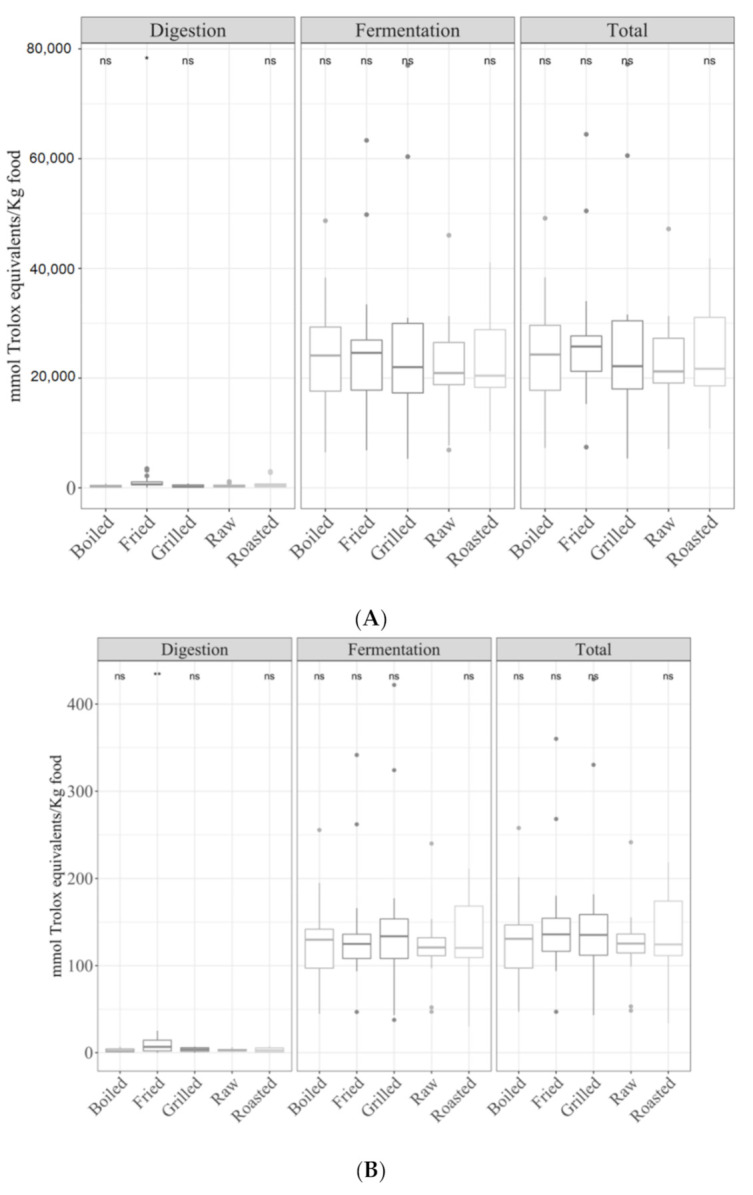

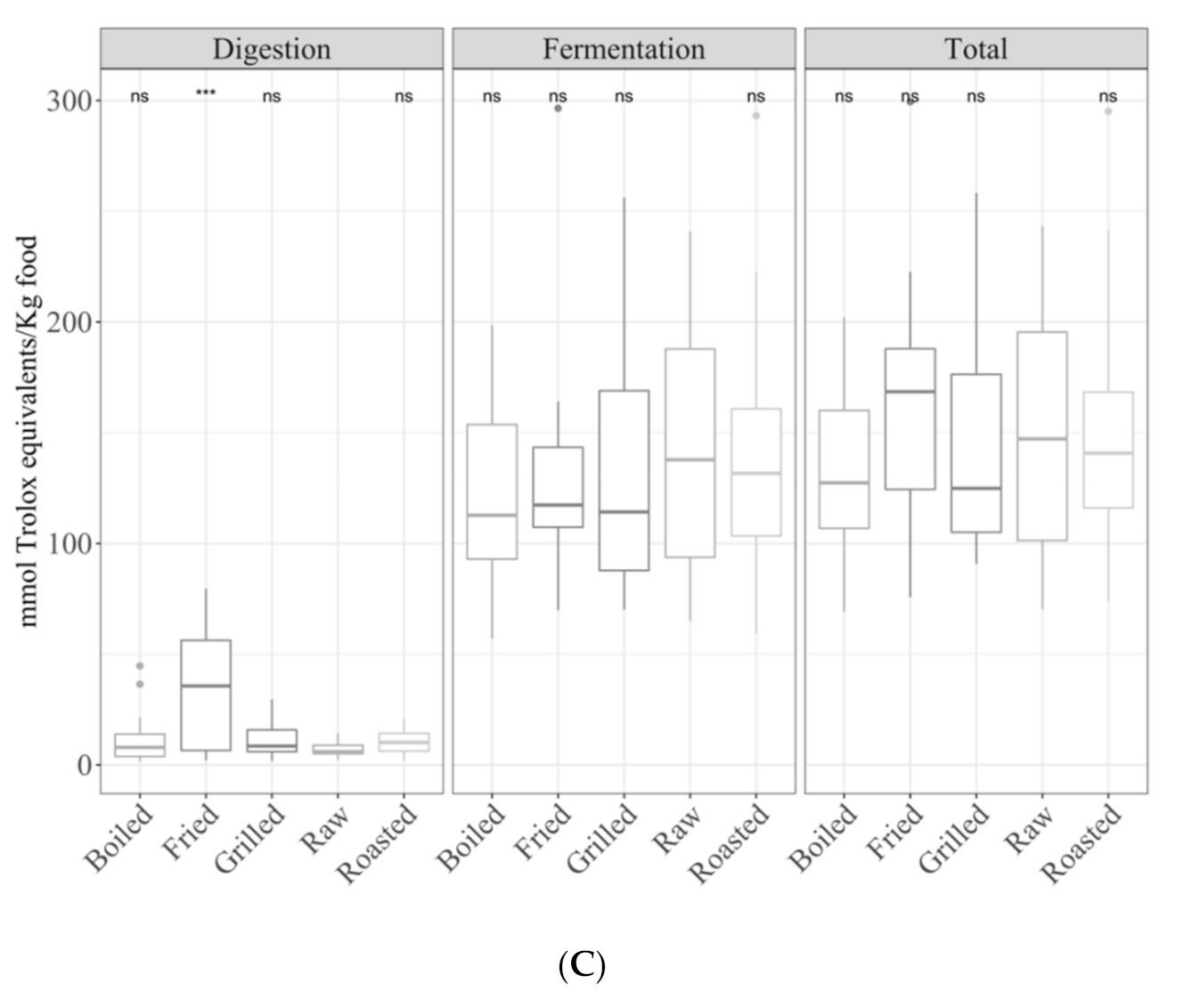
Antioxidant capacity of digested-fermented vegetables depending on the type of cooking technique ((**A**) for TEAC_DPPH_, (**B**) Folin–Ciocalteu and (**C**) TEAC_FRAP_). Statistical analysis was performed via ANOVA using raw vegetables as the reference group. Statistic labels: *: *p <* 0.05, **: *p <* 0.01, ***: *p <* 0.001, ns: not significant.

The analysis of the effect of cooking techniques on vegetables was interesting since these types of foods can be eaten both raw or heat-treated. In the case of the digestion fraction, for the TEAC_DPPH_ (Figure 8A), Folin–Ciocalteu (Figure 8B) and TEAC_FRAP_ (Figure 8C) methods, fried vegetables were more antioxidant than raw vegetables (ANOVA paired comparisons, *p <* 0.05). No statistically significant differences were observed for either the fermented fraction or total antioxidant capacity, although after-fermentation values were always much higher than those obtained for the digested fraction. 

**Figure 9:**
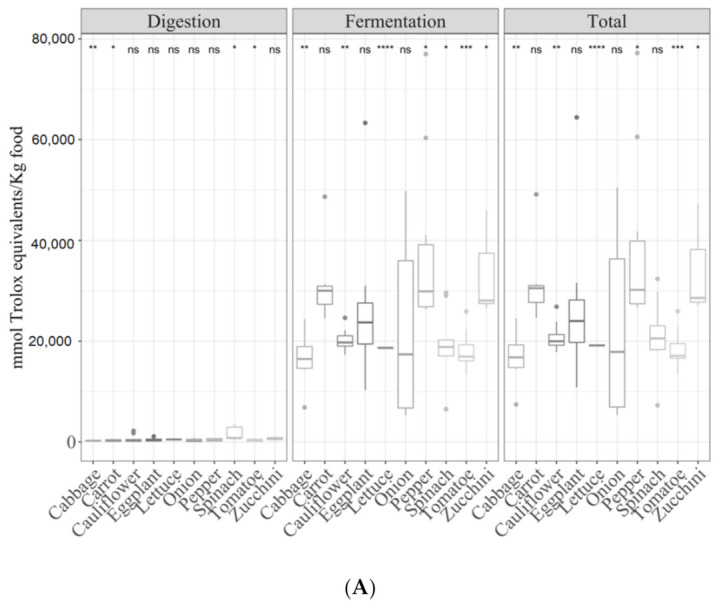

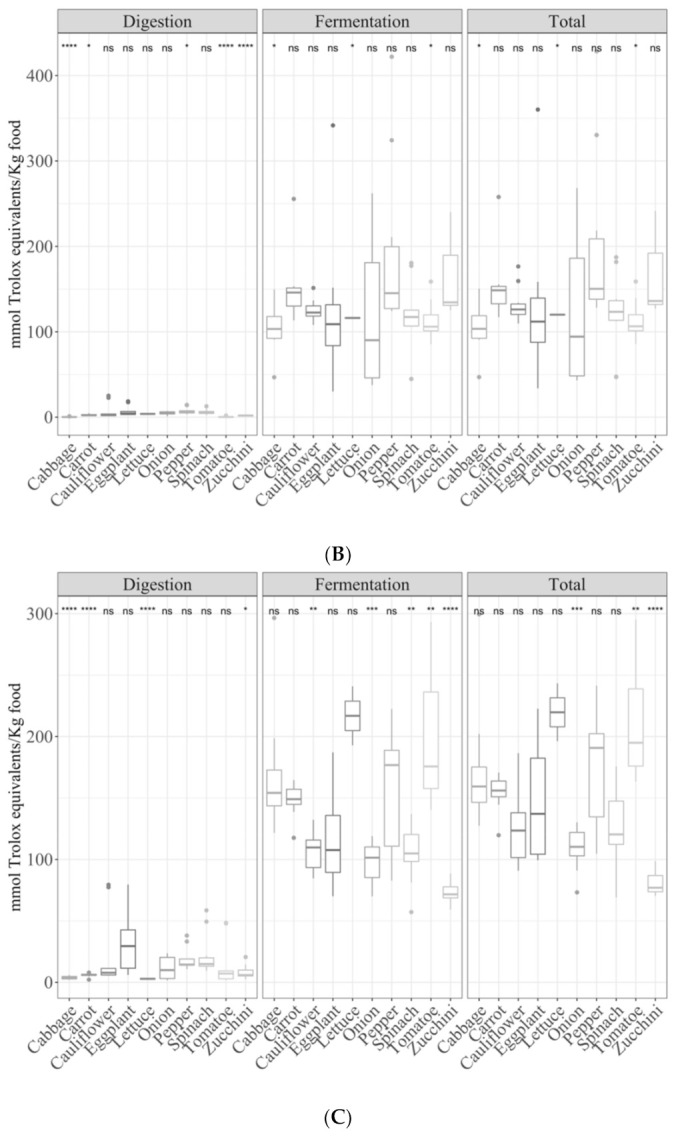
Antioxidant capacity of digested-fermented vegetables depending on sample type ((**A**) for TEAC_DPPH_, (**B**) for Folin–Ciocalteu and (**C**) for TEAC_FRAP_). Statistical analysis was performed via ANOVA using the mean of all food groups as the reference group. Statistic labels: *: *p <* 0.05, **: *p <* 0.01, ***: *p <* 0.001, ****: *p <* 0.0001, ns: not significant.

Ten different vegetables were individually studied (cabbage, carrot, cauliflower, eggplant, lettuce, onion, pepper, spinach, tomato and zucchini). Overall, the antioxidant capacity of the digested fractions across vegetables was similar and not significant (*p <* 0.05) differences were found between most, regardless of the antioxidant assay used (Figure 9A–C). Only cabbage, carrot and zucchini were less antioxidant (*p <* 0.05) than the others. For the fermented fraction and total antioxidant capacity vegetables behaved differently depending on the antioxidant capacity method assessed, and no clear tendency was observed. 

## 4. Discussion 

It is known that heat treatment affects the antioxidant capacity of foods [5,6,9]. In our study, we found that the effect of the cooking technique strongly depends on the antioxidant capacity method used, which agrees with previous results [20]. The different cooking techniques (boiling, grilling, roasting frying and toasting) maintained or increased the total antioxidant capacity of the raw plant foods, suggesting that different antioxidant compounds are generated by thermal processing during the Maillard reaction [21,22,23,24,25,26] or that more antioxidant compounds are released by cell breakage [27,28,29]. On the other hand, the total antioxidant capacity of raw samples was similar to some thermal processes, suggesting that it is not necessary to cook certain foods to achieve the extraction of their antioxidant substances, as suggested by other authors [21,22,23,24]. 

In general, the antioxidant capacity of the fermented fraction is much higher (from 80 to 98% of the total antioxidant capacity), potentially due to the essential role that the gut microbiota plays in the release of antioxidant compounds from the undigested food matrix [12,29]. In fact, antioxidant capacity values obtained for digested and fermented foods are higher than those of foods not submitted to these processes [30]. This reinforces the beneficial effect of digestion and fermentation in the release and transformation of bioactive compounds.

The highest total antioxidant capacity was found in cocoa and the lowest in alcoholic drinks. Cocoa is rich in phenolic compounds, substances with a high antioxidant capacity [31], which explains the larger values obtained regardless of the assay, corroborating previous studies [13,29]. The antioxidant capacity of alcoholic drinks was very low, but other liquid foods (coffee and oils) were highly antioxidant, in line with other papers [31,32,33] even as a source of lipophilic antioxidant compounds [34].

In cereals, biscuits and breakfast cereals were the most antioxidant foods, potentially due to the generation of melanoidins during thermal processing [22,32]. In the case of rice and pasta, they showed low antioxidant capacity but with values similar to those described above [35]. Such a low antioxidant capacity could be related to the loss of antioxidant compounds in cooking water and also to a low-intensity thermal–treatment, which does not favor the development of antioxidant compounds derived from the Maillard reaction and caramelization [29,32]. Frying increased the antioxidant capacity of bread, potentially due to the enrichment with olive oil. However, in fruits and vegetables, the antioxidant capacity was quite homogeneous, with no differences among cooking techniques. 

The antioxidant capacity of a given food could be of interest in regard to its shelf life; higher antioxidant capacity means lower oxidative degradation. However, much attention is drawn to human health. Therefore, it is interesting to calculate the contribution of each kind of food to the daily antioxidant intake in a regular diet. Accordingly, Table 1 shows the contribution of plant foods to the daily antioxidant and total phenolic intake in the Spanish diet. Saura-Calixto and Goñi [19] calculated a total antioxidant intake of 6014 µmol Trolox equivalents/d according to the FRAP method, which was used as a reference (100% total antioxidant capacity) to compare our results. Taking into account the daily intake of each food item/group in Spain [17], the daily mean intake ranged from 296 to 22,423 mol Trolox equivalents/d (coffee and cereals, respectively), which in turn is a contribution of 4.92–373% of the total antioxidant capacity of the diet. A more realistic approach can be obtained by using serving size [18] to calculate the intake of antioxidant capacity per serving (Table 1). With such an approach, the daily intake of antioxidant capacity ranges from 348 to 31,253 µmol Trolox equivalents/d for oils and tubers, respectively. In turn, this realistic approach reaches up to 520% of the daily antioxidant capacity intake calculated [19]. Such high daily intake of antioxidant capacity could be explained taking into account that former calculations performed by Saura-Calixto and Goñi were done with the antioxidant extraction method, which does not take into account the effects of digestion and fermentation in the release of antioxidant compounds. 

## 5. Conclusions

In conclusion, this study reinforces the concept that plant foods are a great source of antioxidant compounds for human beings. After *in vitro* digestion and fermentation, cocoa and legumes stand out among all foods for their antioxidant capacity. In the group of fruits, olives and bananas were the most relevant, whereas lettuce and pepper were the most antioxidant foods in the vegetables group. In addition, based on the results included in this paper, the antioxidant capacity of plant foods has been underestimated in the last decades since the key role of the gastrointestinal system on the release and transformation of antioxidant molecules was not taken into consideration. Therefore, future studies should be conducted including this new approach to test the physiological transformation of foods to calculate their contribution to the daily antioxidant capacity intake. 

## Figures and Tables

**Table 1:** Contribution of plant foods consumption to the daily antioxidant capacity (AOX) in the Spanish diet.

Food type	Analytical Assay	AOX Daily Intake ^1^ (µmol Trolox/d)	AOX Serving Intake ^2^ (µmol Trolox/serving)	Mean Contribution to Daily Antioxidant Capacity Intake (%) ^3^	Mean Contribution to Daily Antioxidant Capacity Intake Per Serving (%) ^3^
Alcoholic drinks	FRAP	1228	3649	20.4	60.7
Cereals	FRAP	22,423	6343	373	106
Cocoa	FRAP	909	6888	15.1	115
Coffee	FRAP	296	1426	4.92	23.7
Fruits	FRAP	19,130	22,230	318	370
Legumes	FRAP	1250	16,655	20.8	277
Nuts	FRAP	1257	5808	20.9	96.6
Oils	FRAP	1157	348	19.2	5.8
Tubers	FRAP	14,804	31,253	246	520
Vegetables	FRAP	15,336	16,001	255	266

^1^ Considering consumption for a whole year; ^2^ Considering the intake of 1 serving; ^3^ Considering the data of Saura-Calixto and Goñi [19].

## Supplementary Materials

The following are available online at https://www.mdpi.com/2076-3921/9/12/1312/s1, Supplemental Table S1: Vegetables and cooking conditions, Supplemental Table S2: Antioxidant capacity of *in vitro* digested-fermented vegetables depending on the cooking method, Supplemental Table S3: Antioxidant capacity of *in vitro* digested-fermented vegetables depending on the vegetables group. Figure S1: Linear correlations between the antioxidant capacity of plant foods.
