# Supplementary material for: Effect of Cooking Methods on the Antioxidant Capacity of Plant Foods Submitted to In Vitro Digestion–Fermentation
Source: Antioxidants (Basel). 2020 Dec 21;9(12):1312. doi: 10.3390/antiox9121312 (PMC7767424; doi:10.3390/antiox9121312)

Effect of cooking methods on the antioxidant capacity of plant foods submitted to in vitro digestion-fermentation

SUPPLEMENTAL INFORMATION

Supporting information description

Supplemental Table S1. Plant foods and cooking conditions.

Supplemental Table S2. Antioxidant capacity of *in vitro* digested-fermented plant foods depending on the cooking method.

Supplemental Table S3. Antioxidant capacity of *in vitro* digested-fermented plant foods depending on the group.

Supplemental Table S4. Antioxidant capacity of *in vitro* digested-fermented cereals depending on the cooking method.

Supplemental Table S5. Antioxidant capacity of *in vitro* digested-fermented cereals depending on the cereal type.

Supplemental Table S6. Antioxidant capacity of *in vitro* digested-fermented fruits depending on the cooking method.

Supplemental Table S7. Antioxidant capacity of *in vitro* digested-fermented cereals depending on the fruit type.

Supplemental Table S8. Antioxidant capacity of *in vitro* digested-fermented vegetables depending on the cooking method.

Supplemental Table S9. Antioxidant capacity of *in vitro* digested-fermented cereals depending on the vegetable type.

Supplemental Figure S1. Linear correlations between the antioxidant capacity of plant foods.

Supplemental Table S1. Plant foods and cooking conditions.

Group	Sample name	Cooking method
Alcoholic	Beer	Brewed
Alcoholic	Red wine	Brewed
Cereals	Biscuits	Raw
Cereals	Whole grain biscuits	Raw
Cereals	Bread	Fried
Cereals	Bread	Raw
Cereals	Bread	Toasted
Cereals	Whole grain bread	Fried
Cereals	Whole grain bread	Raw
Cereals	Whole grain bread	Toasted
Cereals	Breakfast cereals	Raw
Cereals	Whole grgain breakfast cereals	Raw
Cereals	Penne (Pasta)	Boiled
Cereals	Whole grain penne	Boiled
Cereals	Rice (Longo)	Boiled
Cereals	Whole grain rice (Longo)	Boiled
Cocoa	Dark Chocolate	Raw
Cocoa	Nutella	Raw
Coffee	Coffee	Brewed
Coffee	Instant coffee	Brewed
Fruits	Apple	Fried
Fruits	Apple	Grilled
Fruits	Apple	Raw
Fruits	Banana	Fried
Fruits	Banana	Grilled
Fruits	Banana	Raw
Fruits	Banana	Roasted
Fruits	Grapes	Fried
Fruits	Grapes	Grilled
Fruits	Grapes	Raw
Fruits	Grapes	Roasted
Fruits	Olives	Raw
Fruits	Orange	Fried
Fruits	Orange	Grilled
Fruits	Orange	Raw
Fruits	Orange	Roasted

Fruits	Peach	Fried
Fruits	Peach	Raw
Fruits	Peach	Roasted
Fruits	Plum	Fried
Fruits	Plum	Grilled
Fruits	Plum	Raw
Fruits	Plum	Roasted
Legumes	Beans (Kidney)	Boiled
Legumes	Beans (Kidney)	Grilled
Legumes	Kidney beans	Roasted
Legumes	Lentils	Boiled
Legumes	Lentils	Grilled
Legumes	Lentils	Roasted
Nuts	Nut mix	Roasted
Nuts	Nut mixture	Fried
Nuts	Nut mixture	Raw
Nuts	Peanuts	Boiled
Nuts	Peanuts	Grilled
Nuts	Peanuts	Roasted
Oils	Olive oil	Fried
Oils	Olive oil	Raw
Oils	Sunflower oil	Fried
Oils	Sunflower oil	Raw
Tubers	Potato	Boiled
Tubers	Potato	Fried
Tubers	Sweet potato	Boiled
Tubers	Sweet potato	Fried
Vegetables	Cabbage	Boiled
Vegetables	Cabbage	Fried
Vegetables	Cabbage	Grilled
Vegetables	Cabbage	Roasted
Vegetables	Carrot	Boiled
Vegetables	Carrot	Fried
Vegetables	Carrot	Grilled
Vegetables	Carrot	Raw
Vegetables	Cauliflower	Boiled
Vegetables	Cauliflower	Fried
Vegetables	Cauliflower	Grilled
Vegetables	Cauliflower	Raw
Vegetables	Cauliflower	Roasted

Vegetables	Eggplant	Boiled
Vegetables	Eggplant	Fried
Vegetables	Eggplant	Grilled
Vegetables	Eggplant	Raw
Vegetables	Eggplant	Roasted
Vegetables	Lettuce	Raw
Vegetables	Onion	Boiled
Vegetables	Onion	Fried
Vegetables	Onion	Grilled
Vegetables	Onion	Raw
Vegetables	Pepper	Boiled
Vegetables	Pepper	Fried
Vegetables	Pepper	Grilled
Vegetables	Pepper	Raw
Vegetables	Pepper	Roasted
Vegetables	Spinach	Boiled
Vegetables	Spinach	Fried
Vegetables	Spinach	Grilled
Vegetables	Spinach	Raw
Vegetables	Spinach	Roasted
Vegetables	Tomato	Boiled
Vegetables	Tomato	Fried
Vegetables	Tomato	Grilled
Vegetables	Tomato	Raw
Vegetables	Tomato	Roasted
Vegetables	Zucchini	Boiled
Vegetables	Zucchini	Fried
Vegetables	Zucchini	Raw
Vegetables	Zucchini	Roasted

Supplemental Table S2. Antioxidant capacity of *in vitro* digested-fermented plant foods depending on the cooking method.

Cooking technique	TEAC$_{DPPH}$ (μmol Trolox/g)		
	**Digested fraction**	**Fermented fraction**	**Total antioxidant capacity**
Boiled	12.6 ± 11.7^{a}	121 ± 38.4^{a}	134 ± 41.2^{a}
Brewed	31.9 ± 15.2^{a,b}	110 ± 63.9^{a}	141 ± 56.0^{a,b}
Fried	29.3 ± 17.1^{b}	134 ± 49.0^{a}	163 ± 58.6^{a,b}
Grilled	17.3 ± 15.2^{a,b}	155 ± 57.4^{a}	172 ± 59.8^{b}
Raw	19.5 ± 17.2^{a,b}	157 ± 71.2^{a}	176 ± 78.4^{a,b}
Roasted	19.3 ± 14.7^{a,b}	153 ± 55.7^{a}	173 ± 62.1^{a,b}
Toasted	1.09 ± 0.83^{a}	109 ± 21.6^{a}	110 ± 22.2^{a,b}
	**Folin-Ciocalteu (mg gallic acid/Kg)**		
	**Digested fraction**	**Fermented fraction**	**Total antioxidant capacity**
Boiled	552 ± 432^{a}	27343 ± 12142^{b}	149 ± 29.0^{b}
Brewed	691 ± 442^{a,b}	2639 ± 763^{a}	11.6 ± 4.76^{a}
Fried	916 ± 753^{a,b}	23154 ± 12815^{b}	24070 ± 12762^{b}
Grilled	520 ± 472^{a}	29271 ± 17069^{b}	29791 ± 17227^{b}
Raw	840 ± 960^{a,b}	25149 ± 13037^{b}	25988 ± 13309^{b}
Roasted	1447 ± 1686^{b}	28849 ± 15322^{b}	30295 ± 16180^{b}
Toasted	3537 ± 268^{c}	16574 ± 5626^{b}	20110 ± 5562^{b}
	**TEAC$_{FRAP}$ (μmol Trolox/g)**		
	**Digested fraction**	**Fermented fraction**	**Total antioxidant capacity**
Boiled	2.05 ± 2.05^{a}	148 ± 59.3^{b}	150 ± 60.0^{b}
Brewed	21.1 ± 16.5^{b}	22.0 ± 4.49^{a}	43.2 ± 18.1^{a}
Fried	9.19 ± 7.64^{c}	130 ± 65.3^{b}	139 ± 67.3^{b}
Grilled	3.35 ± 2.85^{a,c}	165 ± 88.0^{b}	168 ± 88.7^{b}
Raw	9.19 ± 13.1^{d}	144 ± 66.7^{b}	153 ± 70.9^{b}
Roasted	5.00 ± 6.02^{a,c,d}	162 ± 83.3^{b}	167 ± 86.3^{b}
Toasted	4.89 ± 0.96^{a,c,d}	104 ± 33.1^{b}	109 ± 33.5^{b}

Supplemental Table S3. Antioxidant capacity of *in vitro* digested-fermented plant foods depending on the group.

Food	$TEAC_{DPPH}$ (µmol Trolox/g)		
	**Digested fraction**	**Fermented fraction**	**Total antioxidant capacity**
Alcoholic drinks	28.4 ± 21.8[a,b]	75.7 ± 77.8[a,c]	104 ± 57.0[a,c]
Cereals	11.2 ± 13.6[a]	140 ± 67.3[a,c]	151 ± 76.8[a,c]
Cocoa	40.2 ± 16.4[a,b]	264 ± 33.1[b]	305 ± 25.8[b]
Coffee	35.5 ± 5.31[a,b]	143 ± 20.1[a,c]	179 ± 18.2[a,c]
Fruits	27.0 ± 21.3[b]	156 ± 44.3[a,c]	183 ± 54.8[a,c]
Legumes	19.3 ± 14.7[b]	187 ± 58.4[b]	229 ± 61.5[b]
Nuts	1.09 ± 0.83[a]	143 ± 52.3[a,c]	157 ± 47.1[a,c]
Oils	41.9 ± 8.01[a,b]	84.1 ± 68.9[a]	105 ± 70.5[a]
Tubers	14.2 ± 8.53[a]	140 ± 18.1[a,c]	148 ± 17.3[a,c]
Vegetables	21.4 ± 15.1[a]	132 ± 51.8[a,c]	148 ± 51.7[a,c]
Mean	*24.4 ± 13.5*	*147 ± 49.2*	*171 ± 48.1*
	**Folin-Ciocalteu (mg gallic acid/Kg)**		
	**Digested fraction**	**Fermented fraction**	**Total antioxidant capacity**
Alcoholic drinks	360 ± 100[a,c]	2313 ± 358[a,c]	2673 ± 441[a,c]
Cereals	1395 ± 955[a]	23976 ± 10725[c]	25371 ± 10640[c]
Cocoa	3382 ± 1475[b]	35753 ± 3428[b]	39125 ± 3752[b]
Coffee	1020 ± 393[a,c]	2964 ± 974[a,c]	3985 ± 1189[a,c]
Fruits	466 ± 351[a]	26470 ± 13442[c]	26937 ± 13453[c]
Legumes	1655 ± 1222[a,c]	42451 ± 13380[a,c]	44116 ± 13837[a,c]
Nuts	2686 ± 167[b]	31817 ± 17443[b]	34504 ± 18034[b]
Oils	766 ± 498[a,c]	2131 ± 621[a,c]	2898 ± 615[a,c]
Tubers	300 ± 101[a,c]	35755 ± 5265[a,c]	35954 ± 5181[a,c]
Vegetables	570 ± 679[a,c]	24544 ± 12333[a,c]	25115 ± 12375[a,c]
Mean	*1260 ± 745*	*22809 ± 7797*	*24069 ± 7952*
	**$TEAC_{FRAP}$ (µmol Trolox/g)**		
	**Digested fraction**	**Fermented fraction**	**Total antioxidant capacity**
Alcoholic drinks	7.04 ± 5.95[a]	20.1 ± 2.11[a]	27.1 ± 6.03[a]
Cereals	6.27 ± 5.31[a]	147 ± 63.0[a]	154 ± 65.2[a]
Cocoa	42.1 ± 23.5[b]	217 ± 20.2[b]	259 ± 32.3[b]
Coffee	35.2 ± 8.26[b]	23.9 ± 5.72[b]	59.1 ± 6.87[b]
Fruits	4.34 ± 3.83[a]	151 ± 67.7[a]	155 ± 69.0[a]
Legumes	6.54 ± 3.79[a]	231 ± 70.1[a]	238 ± 70.8[a]
Nuts	15.6 ± 16.4[a]	178 ± 85.8[a]	194 ± 78.7[a]
Oils	6.54 ± 4.95[a]	28.2 ± 12.8[a]	34.8 ± 8.81[a]
Tubers	4.80 ± 0.95[a]	179 ± 30.9[a]	184 ± 30.5[a]
Vegetables	4.31 ± 4.85[a]	133 ± 62.8[a]	137 ± 64.2[a]
Mean	*13.3 ± 7.78*	*131 ± 42.1*	*144 ± 43.3*

Supplemental Table S4. Antioxidant capacity of *in vitro* digested-fermented cereals depending on the cooking method.

Cereals

Cooking technique	DPPH (mmol Trolox equivalents/Kg)		
	**Digested fraction**	**Fermented fraction**	**Total antioxidant capacity**
Boiled	7.38 ± 5.98[a]	94.0 ± 25.7[a]	101 ± 27.5[a]
Fried	5.27 ± 1.77[a]	112 ± 27.7[a]	117 ± 26.3[a]
Raw	19.0 ± 17.4[b]	190 ± 73.9[b]	209 ± 84.8[b]
Toasted	1.09 ± 0.83[a]	109 ± 21.6[a]	110 ± 22.2[a]
	**Folin-Ciocalteu (mg gallic acid/Kg)**		
	**Digested fraction**	**Fermented fraction**	**Total antioxidant capacity**
Boiled	731 ± 207[a]	18738 ± 5453[a]	19468 ± 5338[a]
Fried	1137 ± 158[a,b,c]	17706 ± 466[a]	18842 ± 332[a]
Raw	1210 ± 368[b]	32026 ± 11394[b]	33236 ± 11529[b]
Toasted	3537 ± 268[c]	16574 ± 5626[a]	20110 ± 5562[a]
	**FRAP (mmol Trolox equivalents/Kg)**		
	**Digested fraction**	**Fermented fraction**	**Total antioxidant capacity**
Boiled	0.23 ± 0.16[a]	117 ± 32.1[a]	117 ± 32.0[a]
Fried	10.2 ± 4.12[a]	111 ± 2.74[a]	121 ± 1.44[a]
Raw	9.45 ± 4.51[b]	195 ± 67.0[b]	204 ± 67.7[b]
Toasted	4.89 ± 0.96[a]	104 ± 33.1[a]	109 ± 33.5[a]

Supplemental Table S5. Antioxidant capacity of *in vitro* digested-fermented cereals depending on the cereal type.

Sample	DPPH (mmol Trolox equivalents/Kg)		
	**Digested fraction**	**Fermented fraction**	**Total antioxidant capacity**
Biscuits	35.1 ± 10.9[b]	221 ± 7.69[b]	256 ± 6.09[b]
Bread	3.21 ± 2.23[a]	108 ± 35.1[a]	111 ± 35.1[a]
Breakfast cereals	18.6 ± 17.7[a]	247 ± 28.0[a]	266 ± 38.5[b]
Penne (Pasta)	4.02 ± 2.05[a]	75.9 ± 10.4[a]	79.0 ± 11.4[a]
Rice (Longo)	10.7 ± 7.04[a]	113 ± 21.8[a]	124 ± 17.5[b]
Mean	*14.3 ± 7.98*	*153 ± 20.6*	*167 ± 21.7*
	**Folin-Ciocalteu (mg gallic acid/Kg)**		
	**Digested fraction**	**Fermented fraction**	**Total antioxidant capacity**
Biscuits	1420 ± 264[b]	34688 ± 6731[b]	36108 ± 6487[b]
Bread	1896 ± 1224[a]	19561 ± 8665[a]	21457 ± 8385[a]
Breakfast cereals	1193 ± 554[a]	36987 ± 10737[b]	38180 ± 11221[b]
Penne (Pasta)	884 ± 84.5[a]	17043 ± 2294[a]	17927 ± 2311[a]
Rice (Longo)	578 ± 173[a]	20432 ± 7514[b]	21010 ± 7403[b]
Mean	*1194 ± 460*	*25742 ± 7188*	*26936 ± 7161*
	**FRAP (mmol Trolox equivalents/Kg)**		
	**Digested fraction**	**Fermented fraction**	**Total antioxidant capacity**
Biscuits	14.6 ± 2.54[b]	210 ± 39.6[b]	225 ± 37.5[b]
Bread	7.20 ± 3.42[a]	121 ± 51.0[a]	129 ± 50.5[a]
Breakfast cereals	7.26 ± 3.13[a]	224 ± 63.1[b]	231 ± 64.8[b]
Penne (Pasta)	0.31 ± 0.14[a]	107 ± 13.5[a]	107 ± 14.5[a]
Rice (Longo)	0.16 ± 0.17[a]	127 ± 44.2[b]	127 ± 44.2[b]
Mean	*5.90 ± 1.88*	*158 ± 42.3*	*164 ± 42.1*

Supplemental Table S6. Antioxidant capacity of *in vitro* digested-fermented fruits depending on the cooking method.

Fruits

Cooking technique	DPPH (mmol Trolox equivalents/Kg)		
	**Digested fraction**	**Fermented fraction**	**Total antioxidant capacity**
Fried	40.8 ± 27.3[a]	163 ± 40.6[a]	204 ± 54.3[a]
Grilled	17.8 ± 14.4[a]	152 ± 37.3[a]	170 ± 43.6[a]
Raw	25.1 ± 20.1[a]	150 ± 58.3[a]	175 ± 68.4[a]
Roasted	22.3 ± 13.5[a]	159 ± 36.4[a]	181 ± 43.5[a]
	**FOLIN (mg gallic acid/Kg)**		
	**Digested fraction**	**Fermented fraction**	**Total antioxidant capacity**
Fried	708 ± 451[b]	25833 ± 12108[a]	26541 ± 12215[a]
Grilled	482 ± 362[a]	24348 ± 14326[a]	34830 ± 14302[a]
Raw	251 ± 192[a]	28021 ± 13916[a]	28272 ± 13958[a]
Roasted	462 ± 184[b]	27187 ± 15418[a]	27648 ± 15104[a]
	**FRAP (mmol Trolox equivalents/Kg)**		
	**Digested fraction**	**Fermented fraction**	**Total antioxidant capacity**
Fried	7.79 ± 5.32[a]	152 ± 68.8[a]	160 ± 72.3[a]
Grilled	1.68 ± 1.14[a]	142 ± 71.5[a]	144 ± 71.8[a]
Raw	4.31 ± 2.30[a]	150 ± 51.3[a]	154 ± 51.8[a]
Roasted	2.88 ± 1.87[a]	159 ± 89.7[a]	162 ± 90.5[a]

Supplemental Table S7. Antioxidant capacity of *in vitro* digested-fermented cereals depending on the fruit type.

Sample	DPPH (mmol Trolox equivalents/Kg)		
	**Digested fraction**	**Fermented fraction**	**Total antioxidant capacity**
Apple	8.00 ± 6.02[a]	91.9 ± 18.9[a]	99.9 ± 16.1[a]
Banana	17.3 ± 19.5[a]	141 ± 19.5[a]	158 ± 36.4[a]
Grapes	38.3 ± 17.4[a]	146 ± 9.73[a]	184 ± 17.4[a]
Olives	63.2 ± 0.76[b]	219 ± 7.36[b]	282 ± 8.12[b]
Orange	28.6 ± 28.0[a]	145 ± 42.3[a]	174 ± 55.7[a]
Peach	11.0 ± 3.47[a]	207 ± 34.1[c]	218 ± 32.0[c]
Mean	*29.7 ± 11.5*	*162 ± 23.2*	*192 ± 28.3*
	**Folin-Ciocalteu (mg gallic acid/Kg)**		
	**Digested fraction**	**Fermented fraction**	**Total antioxidant capacity**
Apple	273 ± 239[a]	24309 ± 14353[a]	24582 ± 14379[a]
Banana	479 ± 408[a]	35935 ± 11123[a]	36414 ± 11044[a]
Grapes	332 ± 233[a]	15222 ± 3123[a]	15554 ± 2983[a]
Olives	438 ± 83.5[b]	31701 ± 620[b]	32139 ± 537[b]
Orange	790 ± 553[a]	17433 ± 6189[a]	18223 ± 6691[a]
Peach	443 ± 64.1[c]	17433 ± 6189[c]	18223 ± 6691[c]
Plum	434 ± 217[c]	33051 ± 14934[c]	33485 ± 14978[c]
Mean	*456 ± 257*	*27171 ± 9499*	*27626 ± 9532*
	**FRAP (mmol Trolox equivalents/Kg)**		
	**Digested fraction**	**Fermented fraction**	**Total antioxidant capacity**
Apple	3.02 ± 2.60[a]	143 ± 76.5[a]	146 ± 76.9[a]
Banana	5.55 ± 3.83[a]	181 ± 65.4[a]	186 ± 64.3[a]
Grapes	1.68 ± 1.19[a]	95.9 ± 18.4[a]	97.6 ± 19.3[a]
Olives	9.45 ± 0.23[b]	193 ± 3.64[b]	202 ± 3.87[b]
Orange	4.22 ± 3.86[a]	109 ± 36.4[a]	113 ± 36.9[a]
Peach	2.55 ± 0.55[c]	167 ± 46.3[c]	169 ± 46.0[c]
Mean	*4.77 ± 2.52*	*156 ± 47.8*	*160 ± 48.2*

Supplemental Table S8. Antioxidant capacity of *in vitro* digested-fermented vegetables depending on the cooking method.

Vegetables

Cooking technique	DPPH (mmol Trolox equivalents/Kg)		
	**Digested fraction**	**Fermented fraction**	**Total antioxidant capacity**
Boiled	12.2 ± 11.9[a]	119 ± 37.0[a]	131 ± 35.2[a]
Fried	36.7 ± 29.3[b]	128 ± 49.3[b]	165 ± 55.8[b]
Grilled	11.8 ± 8.72[a]	136 ± 61.0[a]	148 ± 55.7[a]
Raw	7.17 ± 3.98[a]	139 ± 53.5[a]	146 ± 54.0[a]
Roasted	10.7 ± 5.56[a]	141 ± 60.8[a]	151 ± 58.9[a]
	**Folin-Ciocalteu (mg gallic acid/Kg)**		
	**Digested fraction**	**Fermented fraction**	**Total antioxidant capacity**
Boiled	338 ± 232[a]	23906 ± 10870[a]	24244 ± 10826[a]
Fried	1058 ± 981[b]	25962 ± 12926[b]	27020 ± 12861[b]
Grilled	325 ± 247[a]	26757 ± 18324[a]	27082 ± 18328[a]
Raw	381 ± 292[a]	22350 ± 8884[a]	22731 ± 9021[a]
Roasted	766 ± 932[a]	23835 ± 9533[a]	24601 ± 9760[a]
	**FRAP (mmol Trolox equivalents/Kg)**		
	**Digested fraction**	**Fermented fraction**	**Total antioxidant capacity**
Boiled	2.51 ± 2.06[a]	126 ± 50.5[a]	129 ± 51.2[a]
Fried	9.00 ± 8.23[b]	139 ± 65.9[b]	148 ± 69.8[b]
Grilled	3.54 ± 2.35[a]	150 ± 97.4[a]	153 ± 98.1[a]
Raw	2.93 ± 1.51[a]	121 ± 40.7[a]	124 ± 40.8[a]
Roasted	3.25 ± 2.71[a]	131 ± 48.6[a]	134 ± 49.6[a]

Supplemental Table S9. Antioxidant capacity of *in vitro* digested-fermented cereals depending on the vegetable type.

Sample	DPPH (mmol Trolox equivalents/Kg)		
	**Digested fraction**	**Fermented fraction**	**Total antioxidant capacity**
Cabbage	3.92 ± 1.50^{b}	171 ± 55.2^{a}	175 ± 54.6^{a}
Carrot	5.83 ± 1.75^{b}	148 ± 14.6^{a}	154 ± 15.8^{a}
Cauliflower	21.8 ± 29.9^{a}	107 ± 15.8^{a}	129 ± 34.7^{a}
Eggplant	33.5 ± 27.1^{a}	114 ± 35.4^{a}	147 ± 48.9^{a}
Lettuce	2.89 ± 0.61^{a}	217 ± 33.9^{a}	220 ± 33.3^{a}
Onion	11.5 ± 9.56^{a}	96.8 ± 18.4^{a}	108 ± 18.6^{a}
Pepper	18.8 ± 9.37^{a}	155 ± 50.4^{a}	174 ± 45.6^{a}
Spinach	22.3 ± 17.1^{a}	105 ± 23.7^{a}	127 ± 32.4^{a}
Tomato	14.0 ± 18.2^{a}	195 ± 53.3^{a}	209 ± 44.1^{a}
Zucchini	8.58 ± 6.02^{b}	72.7 ± 8.84^{a}	81.3 ± 10.9a
Mean	*14.3 ± 12.1*	*138 ± 31.0*	*152 ± 33.9*
	**Folin-Ciocalteu (mg gallic acid/Kg)**		
	**Digested fraction**	**Fermented fraction**	**Total antioxidant capacity**
Cabbage	291 ± 156^{a}	16407 ± 5079^{a}	16698 ± 4993^{a}
Carrot	292 ± 205^{a}	31018 ± 7609^{a}	31310 ± 7690^{a}
Cauliflower	585 ± 749^{b}	20246 ± 2048^{a}	20831 ± 2681^{a}
Eggplant	491 ± 345^{b}	26306 ± 14288^{a}	26797 ± 14515^{a}
Lettuce	496 ± 78.0^{b}	18669 ± 169^{a}	19165 ± 247^{a}
Onion	320 ± 307^{b}	22069 ± 17712^{a}	22389 ± 17885^{a}
Pepper	420 ± 231^{b}	37885 ± 17271^{a}	38305 ± 17179^{a}
Spinach	1623 ± 1315^{b}	18409 ± 7686^{a}	20032 ± 8119^{a}
Tomato	327 ± 236^{b}	18111 ± 3664^{a}	18438 ± 3621^{a}
Zucchini	656 ± 323^{b}	32357 ± 7307^{a}	33013 ± 7532a
Mean	*550 ± 395*	*24148 ± 8283*	*24698 ± 8446*
	**FRAP (mmol Trolox equivalents/Kg)**		
	**Digested fraction**	**Fermented fraction**	**Total antioxidant capacity**
Cabbage	0.36 ± 0.43^{a}	103 ± 29.9^{a}	103 ± 30.1^{a}
Carrot	2.72 ± 1.02^{a}	152 ± 44.7^{a}	155 ± 44.3^{a}
Cauliflower	6.64 ± 9.17^{b}	125 ± 84.0^{a}	131 ± 84.0^{a}
Eggplant	7.04 ± 5.96^{b}	124 ± 84.0^{a}	131 ± 88.3^{a}
Lettuce	3.87 ± 0.11^{b}	116 ± 0.99^{a}	120 ± 1.11^{a}
Onion	4.74 ± 2.35^{b}	118 ± 85.9^{a}	122 ± 87.2^{a}
Pepper	7.34 ± 3.89^{b}	192 ± 102^{a}	199 ± 101^{a}
Spinach	6.17 ± 3.18^{b}	115 ± 45.2^{a}	121 ± 46.5^{a}
Tomato	0.57 ± 0.73^{b}	113 ± 21.5^{a}	113 ± 21.6^{a}
Zucchini	1.77 ± 0.63^{a}	160 ± 43.0^{a}	161 ± 43.1^{a}
Mean	*4.12 ± 2.75*	*132 ± 46.8*	*136 ± 48.7*

Supplemental Figure S1. Linear correlations between the antioxidant capacity of plant foods.

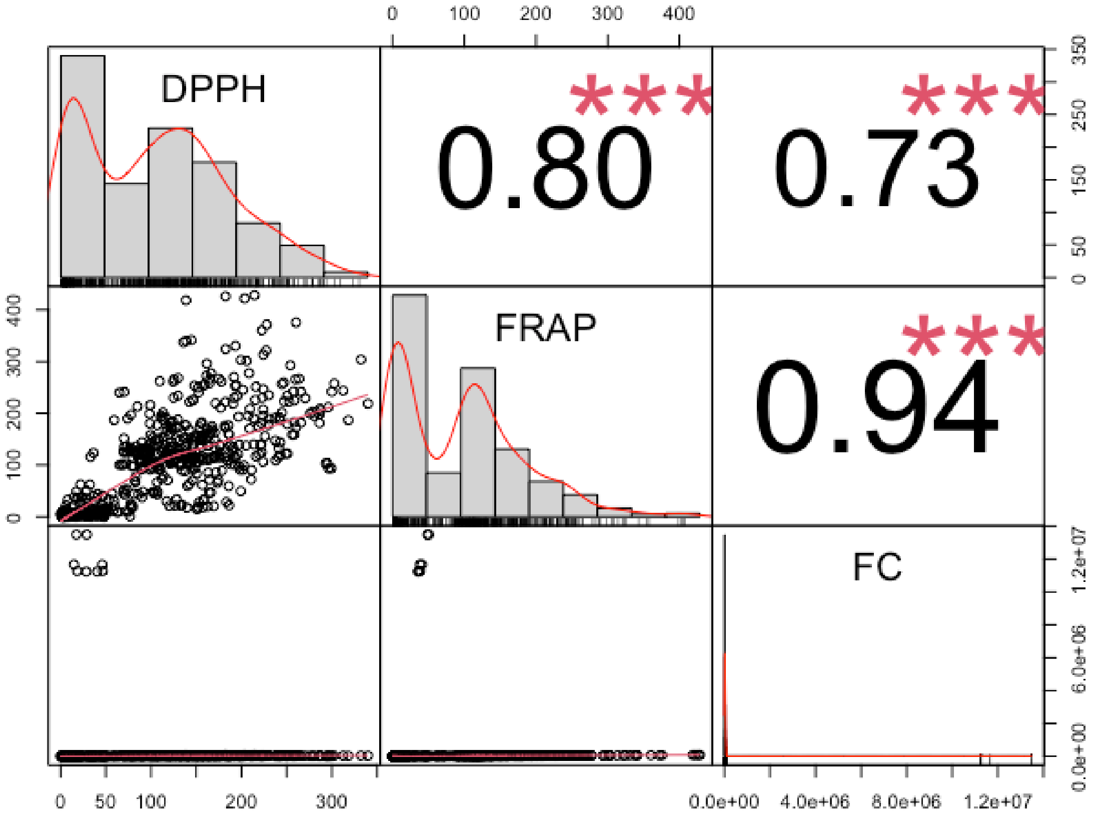

Supplement: Supplementary file 1 [file antioxidants-09-01312-s001.pdf]
